# Similarity in evoked responses does not imply similarity in macroscopic network states

**DOI:** 10.1162/netn_a_00354

**Published:** 2024-04-01

**Authors:** Javier Rasero, Richard Betzel, Amy Isabella Sentis, Thomas E. Kraynak, Peter J. Gianaros, Timothy Verstynen

**Affiliations:** Department of Psychology, Carnegie Mellon University, Pittsburgh, PA, USA; Neuroscience Institute, Carnegie Mellon University, Pittsburgh, PA, USA; School of Data Science, University of Virginia, Charlottesville, VA, USA; Department of Psychological and Brain Sciences, Indiana University, Bloomington, IN, USA; Center for the Neural Basis of Cognition, University of Pittsburgh and Carnegie Mellon University, Pittsburgh, PA, USA; Department of Psychology, University of Pittsburgh, Pittsburgh, PA, USA; Biomedical Engineering, Carnegie Mellon University, Pittsburgh, PA, USA

**Keywords:** Modular brain, Dynamical systems, fMRI, Instantaneous connectivity, Brain activation

## Abstract

It is commonplace in neuroscience to assume that if two tasks activate the same brain areas in the same way, then they are recruiting the same underlying networks. Yet computational theory has shown that the same pattern of activity can emerge from many different underlying network representations. Here we evaluated whether similarity in activation necessarily implies similarity in network architecture by comparing region-wise activation patterns and functional correlation profiles from a large sample of healthy subjects (*N* = 242). Participants performed two executive control tasks known to recruit nearly identical brain areas, the color-word Stroop task and the Multi-Source Interference Task (MSIT). Using a measure of instantaneous functional correlations, based on edge time series, we estimated the task-related networks that differed between incongruent and congruent conditions. We found that the two tasks were much more different in their network profiles than in their evoked activity patterns at different analytical levels, as well as for a wide range of methodological pipelines. Our results reject the notion that having the same activation patterns means two tasks engage the same underlying representations, suggesting that task representations should be independently evaluated at both node and edge (connectivity) levels.

## Author Summary

As a dynamical system, the brain can encode information at the module (e.g., brain regions) or the network level (e.g., connections between brain regions). This means that two tasks can produce the same pattern of activation, but differ in their network profile. Here we tested this using two tasks with largely similar cognitive requirements. Despite producing nearly identical macroscopic activation patterns, the two tasks produced different functional network profiles. These findings confirm prior theoretical work that similarity in task activation does not imply the same similarity in underlying network states.

## INTRODUCTION

The idea of a modular mind (Fodor, 1983), where cognition arises from the interplay between specialized, domain-specific units that represent fundamental cognitive processes, has dominated the cognitive neuroscientific view of the brain since its inception (e.g., Posner, Petersen, Fox, & Raichle, 1988). Here the cognitive “modules” are mapped to unique brain areas that execute specific processes (e.g., detecting specific sound frequencies, estimating value, contracting specific muscle groups; Feinberg & Farah, 2006). Over the last four decades, this modular view of the brain has largely been justified by empirical observations using noninvasive brain imaging methods, like positron emission tomography and functional MRI (fMRI), where experiments and analytical methods were explicitly designed to isolate clusters of regions aligned to certain functional domains, such as vision (e.g., Bihan et al., 1993), control (e.g., Porro et al., 1996), language (e.g., Binder et al., 1997), or affect (e.g., Anders, Lotze, Erb, Grodd, & Birbaumer, 2004).

As a consequence of this early modularist perspective, as well as limitations of early brain imaging technology, a large part of early cognitive neuroscience focused on what was happening at these modules themselves. Many inferences focused on which regions were activated (or deactivated) by specific task conditions. This often led to the implicit assumption that if the same brain regions were activated by two different tasks, then the tasks relied on the same brain networks and, thus, the same underlying cognitive processes. Yet, with the rise of the dynamical systems perspective of the brain (Gelder, 1995; Kelso, 1995), it became increasingly clear that understanding the modules is not enough. In order to understand how tasks are internally represented one must understand the interactions between modules as well. This dynamical systems perspective has gained ground over the past decade in systems neuroscience, where multiunit recording studies have shown that task representations emerge as a low-dimensional manifold of population activity, both within and between brain areas (Churchland et al., 2012; Oby et al., 2019; Russo et al., 2020; Sadtler et al., 2014). This observation at the microscale level extends to observations of macroscopic brain dynamics as well (e.g., Ejaz, Hamada, & Diedrichsen, 2015; Kriegeskorte et al., 2008). With the rise of connectomics (Behrens & Sporns, 2012), the idea of the brain as a dynamical network (Sporns, 2013), where information is also encoded *between* units (Bertolero, Yeo, & D’Esposito, 2015; Crossley et al., 2013; Yeo et al., 2015), has proven to be incredibly useful at explaining both underlying representations and brain-behavior relationships.

One interesting consequence of this network-level perspective is the decoupling of activation patterns from underlying network states: Two tasks can produce the same patterns of activity in the same brain regions, but have fundamentally different underlying network profiles. Indeed, Prinz, Bucher, and Marder (2004) illustrated this concept using a simple three-unit computational model of stomatogastric ganglia in lobsters. Simply by varying the relative connection weights between the three units, the authors showed how multiple underlying network states can be realized as identical patterns of activity at the units themselves. Here we test the predictions of Prinz et al. (2004), at the macroscopic level, by measuring blood oxygen level–dependent (BOLD) dynamics elicited during two response conflict tasks, the color-word Stroop task (Stroop, 1935) and the Multi-Source Interference Task (MSIT; Bush & Shin, 2006). This study builds on previous work exploring the relationship between task activation and functional correlations (Alnæs et al., 2015; Chan, Alhazmi, Park, Savalia, & Wig, 2017; Gratton, Laumann, Gordon, Adeyemo, & Petersen, 2016; Krienen, Yeo, & Buckner, 2014; Newton, Morgan, Rogers, & Gore, 2010; Spadone et al., 2015), but concentrating on these two tasks because they share common computational demands and have overlapping topologies of evoked responses (Sheu, Jennings, & Gianaros, 2012). In a sample of neurologically healthy adults (*N* = 242), we first computed instantaneous functional correlation graphs, using a novel approach that temporally unwraps Pearson correlations to generate time series along edges, representing the internode BOLD signal cofluctuations (Zamani Esfahlani et al., 2020). Then, by means of a general linear model (GLM), we assessed the task-based contributions to the edge time series, quantifying the amount of out-of-sample variability that they contained. We then compared the degree of between-task similarity at the regional activation and connectomic levels.

## RESULTS

### Group-Level Activation Patterns

**Figure 1.**
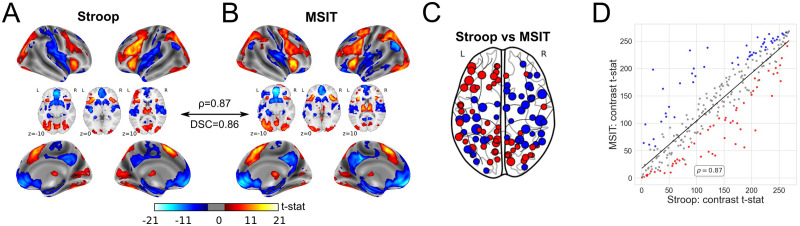
Group-level activation maps. For both Stroop task (A) and MSIT (B), the group-level incongruent-vs.-congruent *t*-statistic maps, at the voxel level for aesthetic reasons. Thus, red colors display higher BOLD activity during incongruent trials compared with congruent trials, whereas blue colors represent the other way around. (C) Using a paired *t* test at *α* = 0.05 (false discovery rate corrected), between-task differences in activation patterns at the region level. Red colors indicate greater incongruent-vs.-congruent values in Stroop than MSIT, and blue colors the opposite. Bigger points correspond to bigger differences between both tasks. (D) The actual correlation comparing the group-level incongruent-vs.-congruent *t*-statistic maps of both tasks. Here, ranks are displayed instead of the actual values, given that the similarity between spatial maps was measured by the Spearman’s correlation. Red and blue colors correspond to the same points displayed in panel C, whereas gray-colored points represent those for which the evoked magnitude response did not significantly differ between the two tasks.

We begin by replicating an exhaustively reported effect (see Sheu et al., 2012, and references therein), namely that the Stroop task and MSIT, both effortful cognitive control tasks, have largely overlapping spatial patterns of evoked activity across the brain, particularly the neocortex (see contrast maps in Figures 1A and 1B). Here such similarity was quantified by a Spearman’s correlation coefficient, *ρ*, between un-thresholded incongruent-vs.-congruent *t*-statistic maps calculated at the region level (voxel-wise estimations with the same region-size spatial smoothing yielded similar values), and a Dice similarity coefficient (DSC), from binarizing these maps as to whether their *t* statistics rejected the null hypothesis at *α* = 0.05 after family-wise (Holm-Bonferroni) error correction. For our group-level activation patterns, the former, *ρ*, was equal to 0.87, and the latter, DSC, was equal to 0.85. As shown in Figures 1A and 1B, increases in brain activity in incongruent trials, with respect to congruent trials, were located in areas typically engaged during the processing of conflictual information and response inhibition, such as the anterior cingulate cortex, anterior insula, parietal cortex, basal ganglia, thalamus, and cerebellum. In contrast, deactivations took place in areas within the ventromedial prefrontal cortex, perigenual anterior cingulate cortex, posterior cingulate cortex, and precuneus, which all comprise the default mode network. As a consequence, these results show that similar cognitive contexts evoke similar patterns of activity across the brain. Nevertheless, both tasks also exhibited substantial differences in the magnitude of their evoked responses, particularly in areas such as the dorsal and medial prefrontal cortex, post- and precentral gyrus, and the precuneus (see Figure 1C). As expected, some of these regions were also the most influential in the spatial correlation between the contrast maps of both tasks (those points further away from the line in Figure 1D).

### Exploration of Cofluctuating Hemodynamics

**Figure 2.**
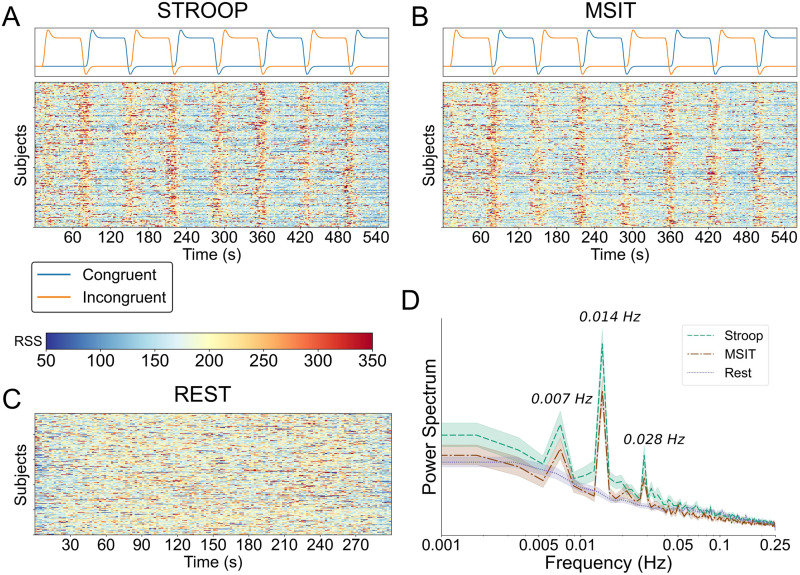
Analysis of the root sum of squares time series. For each subject, the root sum of squares of the edge time series that include the task effects for Stroop task (A), MSIT (B), and resting-state acquisition (C). Their power spectrum (in arbitrary units) using a periodogram (D), averaged across subjects.

For illustrative purposes, we examined the task-related effects on the interregion cofluctuations by computing the root sum of squares across edges at each time frame. It is important to clarify that, for this calculation, parcellated BOLD time series prior to edge time series formation included all task events, in contrast to subsequent analyses. As shown in Figures 2A and 2B, during both tasks moments of high cofluctuations tended to be synchronized across subjects, concentrated mostly around the rest periods separating congruent and incongruent block conditions. In both the congruent and incongruent blocks, there appeared to be a consistent reduction in global functional connectivity, with sporadic and inconsistent periods of brief synchronous activity that qualitatively appear more frequent during incongruent blocks.

In contrast to the task patterns, for the resting-state run, where no external stimulus was presented, we did not see evidence of between-subject synchronization of high amplitude cofluctuations (Figure 2C). However, the overall presence of these brief cofluctuations appears to be qualitatively more frequent in the resting-state run than during either of the two tasks. These results were further confirmed by inspecting the subject-averaged power spectrum of the root sum of squares for the three tasks (Figure 2D). For both Stroop and MSIT, there was an overall increase in power at frequencies consistent with task onsets and offsets.

### Group-Level Functional Correlations

**Figure 3.**
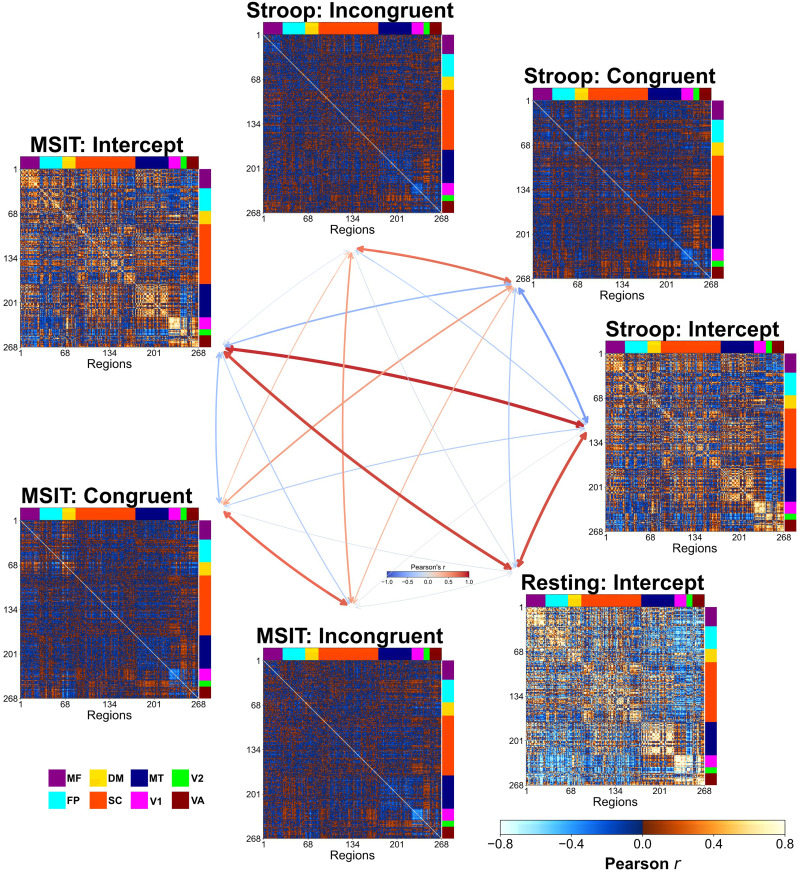
Functional correlation matrices at the group level. For Stroop task, MSIT, and resting-state functional correlation matrices using the intercept, congruent, and incongruent GLM estimations at the group level. Regions (i.e., the rows and columns) have been arranged based on their belonging to a major intrinsic network system (see Methods). In the middle in the form of a graph, the Pearson’s correlation coefficients between the upper-triangular elements of these matrices. MF: medial-frontal; FP: frontoparietal; DM: default mode; SC: subcortical-cerebellum; MT: motor; V1: visual-1; V2: visual-2; VA: visual-association.

We estimated task-related functional correlations using a GLM on the edge time series. Three coefficients (i.e., intercept, congruent, and incongruent) for both Stroop task and MSIT were estimated for each edge, while for resting state a single coefficient per edge was obtained (i.e., intercept only). The resulting group-level network profiles are displayed in Figure 3, where the *t* statistics for each of these coefficients were converted to correlations using the transformation, *r*^2^ = $\frac{t^{2}}{t^{2}+N-1}$ with *N* being the number of subjects.

The first thing to note is that, after accounting for condition effects during the two tasks, we were able to recover the intrinsic brain networks observed during resting-state. The intercept profiles for both Stroop and MSIT had a high degree of similarity to the resting-state profile (*r* = 0.85 and *r* = 0.86, respectively), as well as a high degree of similarity to each other (*r* = 0.94).

On the other hand, a largely different profile emerged during congruent and incongruent conditions in both tasks. These networks showed much lower overall functional correlations, and a shift towards more negative correlations, than the intercept profiles. Despite this difference from the intrinsic networks, the condition-related profiles (i.e., congruent and incongruent) had a decent degree of within-task similarity (*r* = 0.72 for Stroop and *r* = 0.75 for MSIT), demonstrating that both conditions recruit largely consistent networks overall. Less similarity was observed in between-task profiles, whether using within-condition comparisons (*r* = 0.54 for congruent; *r* = 0.83 for incongruent) or between-condition comparisons (Stroop congruent and MSIT incongruent *r* = 0.39, Stroop incongruent and MSIT congruent *r* = 0.31).

Taken together, these results confirm that our method was able to reliably characterize both task and intrinsic (resting) networks, at the group level, using the edge time series.

### Network Profile Differences Between Task Conditions

**Figure 4.**
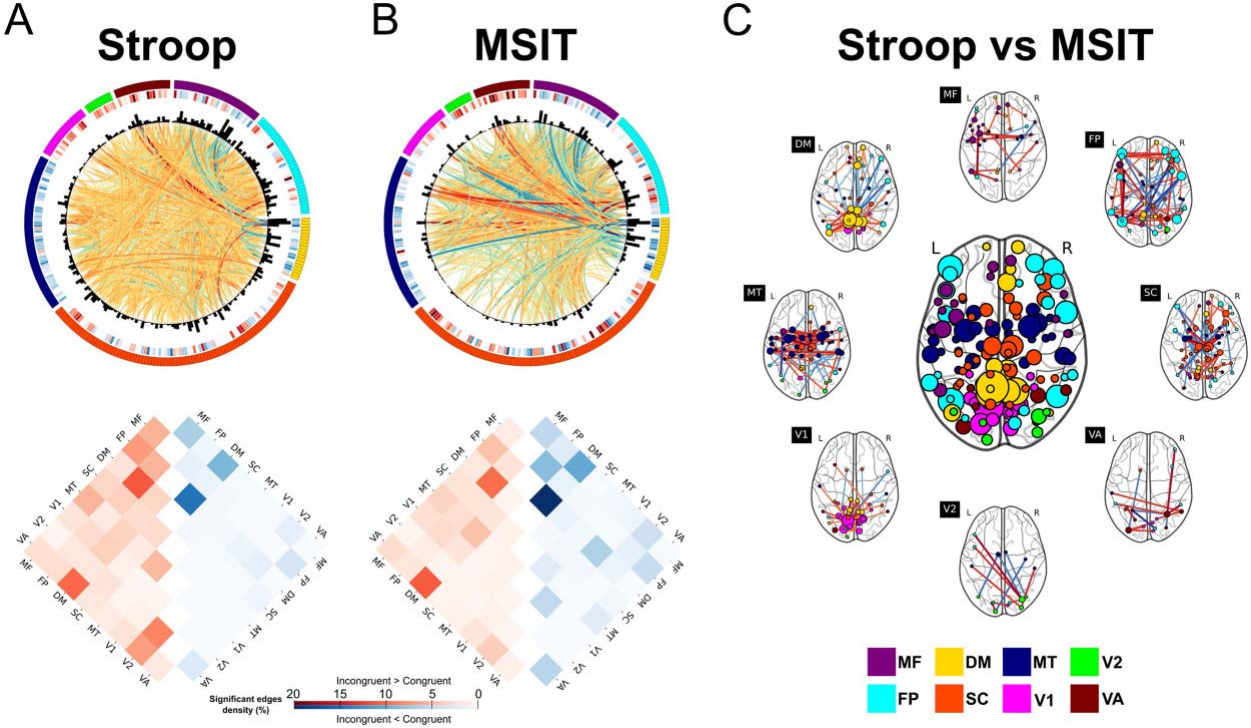
Group-level incongruent-vs.-congruent functional correlation differences. For Stroop task (A) and MSIT (B), on the top side and from outer to inner circular, plots display each region arranged and colored according to the major functional system, their incongruent-vs.-congruent activity at the node level, their degree from the incongruent-vs.-congruent significant edges, and finally the *t* statistic of these edges (red: incongruent > congruent, blue: incongruent < congruent). At both node and edge levels, only significant results (at *α* = 0.05, Bonferroni corrected) are shown. The bottom side shows the number of significant edges within and between major functional connectivity networks, normalized by the total number of edges in each case. (C) Using the significant edges from a paired *t* test at *α* = 0.05 (false discovery rate corrected), between-task differences in incongruent-vs.-congruent functional correlations are shown in region degree (inner brain plot), and with the edge *t* statistics to regions of each major functional system (outer brain plots). MF: medial-frontal; FP: frontoparietal; DM: default mode; SC: subcortical-cerebellum; MT: motor; V1: visual-1; V2: visual-2; VA: visual-association.

The network profiles that emerged as a consequence of conflict processing were quantified at the group level by contrasting subject-level functional correlations from both task conditions. The resulting incongruent-vs.-congruent statistical maps for both tasks are displayed in Figure 4 (top plots, panels A and B), with 1,284 (Stroop task) and 1,042 (MSIT) edges that were significant at *α* = 0.05 after family-wise (Holm-Bonferroni procedure) error correction (red colors denote greater functional correlations during incongruent trials than during congruent trials, and blue colors the opposite). In both cases, network differences were primarily associated with default mode, frontoparietal, medial-frontal, and visual systems, as measured by the average significant edges per region found in those networks. Furthermore, inspecting the sign of these differences (Figure 4, bottom plots in panels A and B), increased functional correlations appeared to be dominated by edges connecting regions of distinct intrinsic major systems, particularly those between the default mode and the frontoparietal and visual-association systems, and medial-frontal areas with the frontoparietal cortex. In contrast, significant decreases in functional correlations during incongruent trials appeared in regions of the same major system, especially those within the default mode and medial-frontal networks.

However, despite the apparent qualitative similarity in network-level responses to congruent and incongruent conditions, the Stroop and MSIT also exhibited key differences. For example, concentrating on the 10% of edges with the largest absolute *t*-statistic values (*N* = 358), the Stroop task contained a significantly greater number of positive (i.e., increased functional correlation during incongruent trials) to negative (i.e., decreased functional correlation during incongruent trials) edges than the MSIT (Fisher’s exact test, odds ratio = 4.10, *p* = 1.17 × 10^−14^). On the other hand, a paired-sample *t* test performed on individual edges revealed that these between-task network differences spanned the entire brain (see Figure 4C), though they prominently expressed in the dorsolateral prefrontal and posterior parietal cortex, both responsible for executive function, as well as in the posterior cingulate cortex, which is strongly implicated during control processes, and the primary visual cortex. As a consequence, these results suggest that the Stroop task and MSIT have substantial differences in their network profiles.

### Comparison of Similarities in Activation Patterns and Network Profiles Between Tasks

**Figure 5.**
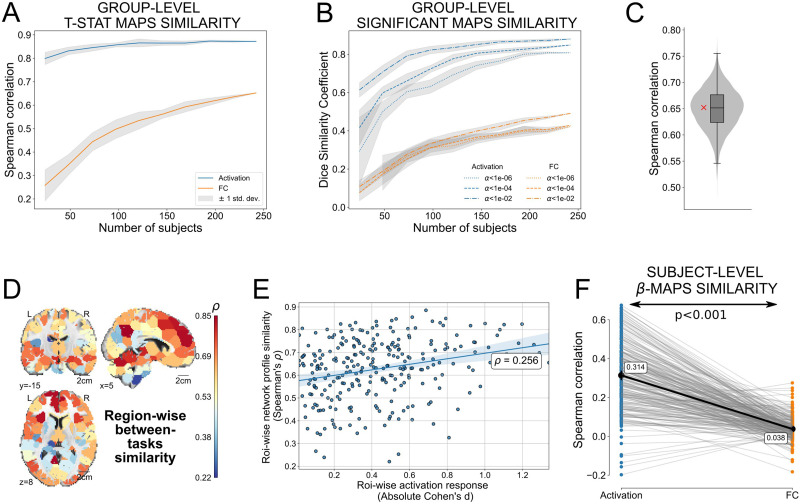
Between-task similarity of activation patterns and network profiles. (A) Spearman’s correlations between tasks from the group-level *t*-statistic incongruent-vs.-congruent maps for both brain activation (blue line) and task-based functional correlations (FC, orange line), varying the number of subjects used for their estimation. Each curve represents the average similarity and the gray area is the standard deviation after repeating 10 times the estimation procedure to consider different subjects. (B) Same as panel A but using the Dice similarity coefficient. Statistical maps were binarized according to whether each *t* statistic was significant under several thresholds of *α*. (C) Distribution of Spearman’s correlations *ρ* between MSIT and Stroop functional correlation profiles from 10,000 subsamples that each randomly selected a subset of edges equal to the number of regions (268). The red cross displays the correlation using the full profiles (i.e., 35,778 edges). (D) Region-wise similarity between tasks, using the whole-brain incongruent-vs.-congruent network profile of each region. (E) These similarity rates per region (y-axis) are plotted versus their activation levels, measured as the average of both tasks’ incongruent-vs.-congruent absolute Cohen’s *d* at the group level. (F) For each subject (a dot in the figure), the Spearman’s correlation between the incongruent-vs.-congruent *β* map of each task for both brain activation (blue points) and task-based functional correlations (orange points). A paired *t* test then quantified the statistical difference between both distributions.

We have previously shown that both Stroop and MSIT elicit largely overlapping patterns of brain activation (*ρ* = 0.87, *DSC* = 0.85; see also Sheu et al., 2012). In contrast, estimated edge-wise responses suggest that both tasks appeared to differ at the network level. Is the lower similarity of network profiles between-task really that different than the similarity in activation patterns? The between-task similarity in incongruent-vs.-congruent network profiles was equal to *ρ* = 0.65 and *DSC* = 0.42 at *α* = 0.05, after family-wise (Holm-Bonferroni) correction, which indeed constitutes a considerable reduction with respect to the aforementioned similarity rates from activation patterns. Furthermore, this reduction became even more evident as the number of subjects decreased (Figure 5A), suggesting that this does not reflect an issue with statistical power in our sample. Also, this effect is largely insensitive to using Spearman’s *ρ* as a similarity measure since the same effect was observed using Dice similarity coefficients at different thresholds (see Figure 5B).

In order to show that this reduction in similarity scores between the incongruent-vs.-congruent functional correlation graphs was not due to correlating a larger number of features from the edges ($\frac{268\times 267}{2}$ = 35,778 edges) than in the activation maps (only 268 components, since these were also considered at the region level), we repeated this calculation taking subsamples (number of subsamples = 10,000) that randomly selected 268 edges in the functional correlation profiles. Across all subsets, we found similar between-task Spearman’s correlation values (0.65 ± 0.04, see Figure 5C) as the one using the full network.

Along similar lines, we explored how the similarity of network profiles was expressed across the brain by correlating, for each region, the whole-brain incongruent-vs.-congruent functional correlation profile (a vector of 267 *t*-statistic values, i.e., we do not consider the diagonal terms in the functional correlation profiles) at the group level of both tasks (see Figure 5D). This analysis showed that there are certain regions, particularly in the superior medial and dorsolateral-frontal gyrus, the precuneus, and the anterior lobe of the cerebellum, that exhibit comparable, and sometimes even greater, similarity values than that from activation patterns. While regions with the largest between-task similarities in activation did tend to have higher degrees of between-task similarity in network profiles (Figure 5E), this association was fairly weak (*ρ* = 0.256), suggesting that our main conclusion would also be reached if one focused exclusively on the subnetwork typically engaged during both Stroop and MSIT.

Since the previous calculation concentrated exclusively on group-level patterns, we also tested whether the same qualitative findings were present at the within-subject level. Specifically, for each individual we correlated, between tasks, the incongruent-vs.-congruent activation maps and functional correlation graphs, using in both cases the *β* estimations (see the sample distributions in Figure 5F). The reason for using the *β* estimations here instead of the *t*-statistic values is that temporal autocorrelations in the time series produced a different number of degrees of freedom across nodes and edges in both tasks, in contrast to the group level, where the degrees of freedom always remained the same (*N* – 1, with *N* the number of subjects). A paired *t* test showed that, as found before with the group-level maps, between-task similarity rates of brain activation maps (< *ρ* > = 0.314, 95% CI [0.292, 0.335]) were higher than those from task-based network differences (< *ρ* > = 0.038, 95% CI [0.031, 0.045]; Cohen’s *d* = 1.584, *p* < 0.001).

The preceding analysis shows how two tasks with similar activation patterns may not have as similar network profiles. One likely explanation for this could be that the correlation ceiling for the former measure (activation) is simply higher than the ceiling for the latter (connectivity), but two individuals more similar in activation patterns may also be more similar in their functional network architecture. In order to rule this out, we conducted, for each task separately, a Mantel test on the upper triangular terms of the distance matrices $d_{ij}^{act}$ and $d_{ij}^{conn}$. This directly tests whether similarity in activation patterns correlates with similarity in network profile at the pairwise subject level. For consistency with the previous analyses, we adopted a Spearman’s correlation–based metric, that is, *d*_*ij*_ = 1 − *ρ*_*ij*_, for defining the distance between any pair of subjects *i* and *j*. We found that these two matrices were not significantly correlated in either of the two tasks (Stroop: *r* = 0.005, 95% CI [−0.123, 0.159]; MSIT: *r* = 0.072, 95% CI [−0.055, 0.204]). Confidence intervals were calculated using 100,000 resamples without replacement and a subsampling ratio of 0.135, following the indications in Balakrishnan, Choe, Singh, Vettel, and Verstynen (2018). These results suggest that the observed difference in similarity rates between activation patterns and network profiles exists beyond any ceiling effect and is persistent even in within-subject comparisons.

We ran several follow-up tests to examine the robustness of all these findings with respect to changes in the analytical pipeline. First, we investigated whether the reduction in between-task similarity in network profiles, compared with evoked responses, was not due to removing the task stimuli prior to calculating the edge time series. Supplementary Figure 1A (see the Supporting Information) shows a mild increase at the subject level when task effects are maintained (< *ρ* > = 0.080, 95% CI [0.071, 0.088]) and it is still significantly lower with respect to activation patterns (Cohen’s *d* = –1.535, *p* < 0.001). Moreover, a similar finding was observed (see Supplementary Figure 1, panel B) when we concentrated exclusively on the regions with the greatest task activation responses (group-level incongruent-vs.-congruent absolute Cohen’s *d* larger than 0.8 in both tasks). Thus the choice of how we regress out task effects prior to building the edge time series does not drive our primary effect of differences in similarity profiles between activation and network profiles.

Subsequently, we tested whether including in our parcellation specific brain structures that are known to be noisier or more susceptible to signal loss, namely the cerebellum and subcortex, might have driven our findings. In order to achieve this, we repeated the subject-level similarity analysis using a Craddock atlas (Craddock, James, Holtzheimer, Hu, & Mayberg, 2012), consisting of 200 regions that did not include the cerebellum, and the Schaefer atlas (Schaefer et al., 2018), comprising 200 cortical regions. Additionally, we considered a combination of 10 ICA-based major areas from Smith et al. (2009), and 7 bilateral subcortical regions from the Harvard-Oxford atlas (thalamus, caudate, putamen, pallidum, hippocampus, amygdala, and accumbens). This customized atlas was included to intentionally test a parcellation that yields a smaller number of edges than the number of nodes in the Shen atlas (268 regions). We found, again, that the choice to use the Shen atlas was not decisive in our primary effect of the differences between activation and network profile similarities (see Supplementary Figure 1, panel C).

Likewise, we wondered whether the reduction in similarity between Stroop and MSIT task-dependent network profiles was influenced by the edge time series approach itself. We tested this possibility by replicating our analyses using a generalized psychophysiological interaction (PPI) model, which is a standard and common framework for assessing task-modulated functional connectivity (see Materials and Methods for details on this model). As Supplementary Figure 1D illustrates, these PPI-based network profiles also showed a reduced similarity between tasks (*ρ* = 0.550, *DSC* = 0.357 at *α* = 0.05 after family-wise error correction) compared with what is observed in the brain activation patterns. In addition, albeit small differences existed, particularly within the motor system, both approaches (edge time series and PPI) appeared to yield fairly similar incongruent-vs.-congruent contrast network profiles in both tasks (*ρ* = 0.743 for Stroop, *ρ* = 0.786 for MSIT).

A compact summary of the between-task activation and network profile similarity values covering debatable methodological choices can be found in Supplementary Figure 2. These comprised how aggressively task stimuli were removed before computing the edge time series, the hemodynamic response function model, whether global signal regression was performed, whether time series at the node and edge levels were standardized in the GLM, and whether prewhitening was applied. We can see that our findings at both the group and the single-subject levels were consistent across all the different methodological setups. This notably included global signal regression, a step that is still controversial in task-effect estimations (Liu, Nalci, & Falahpour, 2017).

Finally, one may argue that the observed differences in between-task similarity degrees are simply a consequence of functional connectivity being inherently noisier than activation measures. Indeed, the signal-to-noise ratio (SNR), defined for our contrast-of-interest as SNR = $\left(\frac{\left|\beta_{inc}-\beta_{con}\right|}{\sigma_{\left(inc-con\right)}}\right)$ and calculated as the median value across nodes/edges, is weak for connectivity (group-level SNR_stroop_ = 0.09, SNR_msit_ = 0.081) and small-to-medium for activation (group-level SNR_stroop_ = 0.38, SNR_msit_ = 0.406). As a consequence, we reran our analysis concentrating exclusively on those nodes and edges with at least a medium SNR at the group level in both tasks (SNR > 0.5). We found that even in this (stringent) scenario with balanced signal-to-noise ranges there remain substantial differences in between-task similarity values at both the group (activation: *ρ* = 0.911, connectivity: *ρ* = 0.775) and the subject levels (activation: < *ρ* > = 0.547, 95% CI [0.524, 0.574], connectivity: < *ρ* > = 0.301, 95% CI [0.27, 0.333]; activation vs. connectivity Cohen’s *d* = 0.819, *p* < 0.001). Thus, signal-to-noise does not appear to be a mediating factor in this effect.

## DISCUSSION

Here we set out to validate previous computational modeling work showing that similarity in patterns of activation do not imply similarity in underlying network states (Goldman, Golowasch, Marder, & Abbott, 2001; Golowasch, Abbott, & Marder, 1999; Prinz et al., 2004; Roffman, Norris, & Calabrese, 2012). Using a GLM framework on instantaneous functional correlation estimates (Faskowitz, Esfahlani, Jo, Sporns, & Betzel, 2020; Zamani Esfahlani et al., 2020), we were able to successfully separate task-free (intrinsic) from task-dependent network contributions, in line with the extensive evidence that task functional correlations are jointly shaped by both intrinsic and evoked network architectures (Cole, Bassett, Power, Braver, & Petersen, 2014). Subsequently, we showed how our two tasks shared a large degree of similarity in activation topology (nodes), but substantially less similarity in network profiles (edges). This difference in task effects at the nodes and edges was confirmed at both group and subject levels, and using two different measures commonly employed for representational similarity analyses. Likewise, this difference between activation and network profiles was replicated after keeping task effects in the edge time series, employing different parcellations, using a different method for estimating task-related functional connectivity (i.e., PPI), exploring a wide array of methodological choices (e.g., including or excluding the brain global signal as a covariate), and balancing signal-to-noise differences. Taken together, these results highlight how similarity in activation does not necessarily imply similarity in underlying network profile, reflecting the fact that the underlying cognitive processes manifest at both the node (voxel or region) and the edge (connectivity) levels.

As pointed out by Prinz et al. (2004), the multiple realizability problem of many network states leading to the same activation pattern poses a challenge when interpreting subject-to-subject differences (Prinz et al., 2004; see also Krakauer, Ghanzanfar, Gomez-Marin, MacIver, & Poeppel, 2017). Indeed, over the past 10 years there has been both increased interest in, and increased pushback against, using task-related fMRI as a means of predicting individual differences in healthy (Gianaros et al., 2020, 2022; Greene, Gao, Scheinost, & Constable, 2018; Jiang et al., 2020; Rosenberg et al., 2016; Tetereva, Li, Deng, Stringaris, & Pat, 2022; Wager et al., 2013) and pathological populations (Andersen, Rayens, Liu, & Smith, 2012; Costafreda et al., 2011; Hart et al., 2014; Just, Cherkassky, Buchweitz, Keller, & Mitchell, 2014; Koch et al., 2015; Mourão-Miranda et al., 2011; Yoon et al., 2012). A fundamental assumption of the statistical tools used in these studies is that if two people are similar in their brain activation they will also be similar in the outcome measure being predicted. Our results show how this fundamental assumption may not always hold: Two people may produce the same pattern of task-related activation, but rely on fundamentally different network-level representations. Indeed, this may help to explain why the effect sizes of brain phenotype studies are so low compared with what would be needed to produce medical-grade diagnostic tools (Marek et al., 2022).

**Figure 6.**
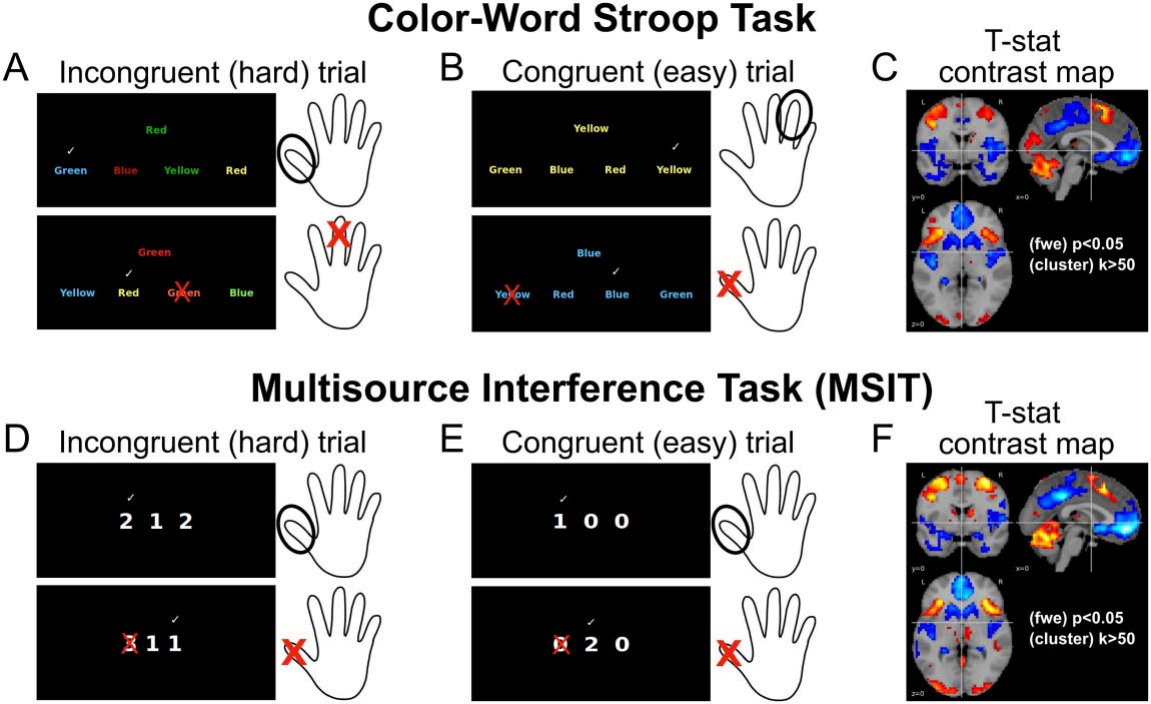
Stroop task and MSIT paradigms and their brain response. For both Stroop task and MSIT, illustration of incongruent (A, D) and congruent trials (B, E). Trials consisted of blocks of 52–60-s duration, interleaved with a 10–17-s fixation block. Contrasting brain activity between incongruent and congruent conditions gives rise to a similar brain response (C, F).

It is worth pointing out that while network profiles do differ more between tasks than activation patterns, we still observed a modest degree of similarity in network profiles across tasks. This is not surprising given the existence of a core functional architecture shared between even markedly different task states (Krienen et al., 2014). In our case, the greatest similarities were found in networks that are reliably associated with sensory processing and motor planning. While motor planning constraints were identical across tasks (i.e., both involved button presses with the same hand and fingers), the visual stimuli were quite different (see Figure 6). This suggests that the between-task dissimilarities in network profiles reflect differences in *how* sensory information is used during action selection, after sensory representations are formed, rather than simple bottom-up effects driven by the stimulus differences between the Stroop task and MSIT. Adding to the other between-task topological differences that we observed, involving mainly regions of the default mode and executive networks, this appears to suggest that greater deviations take place in subnetworks largely associated with higher level cognitive functions.

One natural follow-up question is how the edge time series responses compare with other approaches for addressing task-based networks like PPI. We have shown that, even though PPI arrives at the same conclusion as the edge time series method, the network profiles obtained from both approaches were not perfectly identical. While the edge time series straightforwardly represents measures of (instantaneous) functional correlations, PPI was designed to assess effective connectivity (K. J. Friston, 2011; K. J. Friston et al., 1997). Thus, in order to enable the comparison between both approaches in our study, PPI estimates were symmetrized, so we speculate that part of these differences may come from this operation. A full comparison with other common methods for task-related networks, such as correlational PPI (Fornito, Harrison, Zalesky, & Simons, 2012) or beta series correlations (Rissman, Gazzaley, & D’Esposito, 2004), could both yield interesting differences and show areas of robustness in network profiles. However, this is well beyond the scope of the current project.

While our findings here provide strong evidence in support of the idea that similarity in activation does not imply similarity in network state, it is worth noting some significant limitations. First, all our analyses have been performed at the macroscopic level. As mentioned above, even though evidence suggests that a similar behavior is expected at smaller network scales, future studies should test this *ex profeso*. On a related point, since the correlation matrices become computationally intractable at the voxel level, and in order to maintain both activation and network measures with the same spatial resolution, we opted to perform all analyses at the region level, using a predefined parcellation template. This obviously introduces some degree of anatomically bounded spatial smoothing in the data, which may be contributing to inflating the similarities in both task-related activation and network profiles between tasks. Smoothing would be problematic if we were interested in null hypothesis tests on spatial patterns (Markello & Misic, 2021); however, the analysis used here does not rely on such spatial hypothesis testing. Thus, this region-level approach does not invalidate the main conclusions of our study that similarity in the topology of activation patterns does not perfectly associate with similarity in network architecture.

Finally, one might question whether the BOLD time series first needed to be deconvolved with the hemodynamic response function prior to estimating the edge time series. It has been argued that deconvolution in block-design tasks, like our Stroop task and MSIT, may not be necessary (Di & Biswal, 2017; Di, Zhang, & Biswal, 2021). However, it is important to point out that while changing the choices in the preprocessing and analysis steps may lead to nuanced differences in certain aspects of our results, none of these potential limitations would likely change the primary conclusion we have drawn from our observations.

Regardless of these limitations, our results clearly illustrate that important aspects of task representations are encoded in the associations between regions, which are unique to and complement information reflected in the spatial topology of activation (Chan et al., 2017; Gratton et al., 2016). Indeed, our findings bolster previous work looking at informational connectivity (Coutanche & Thompson-Schill, 2013), which highlights the information value of associations between regions in understanding task representations. This poses significant challenges for interpreting individual differences based on activation patterns alone. Further work should dig deeper into the high-dimensional relationships between localized activation and global connectivity dynamics when trying to understand the nature of representations in the brain.

## MATERIALS AND METHODS

### Participants

We analyzed task and resting-state fMRI data from the Pittsburgh Imaging Project (PIP), which is a registry of behavioral, biological, and neural correlates of cardiovascular disease risk among otherwise healthy community-dwelling adults (aged 30–54 years). Details of this project can be found in the supplementary material of Gianaros et al. (2020). We selected a subset of 242 subjects (female = 119, mean age = 40 ± 6 years, min. age = 30 years, max. age = 51 years) that had full temporal and spatial coverage and exhibited low average motion (mean framewise displacement, estimated using the method in Power, Barnes, Snyder, Schlaggar, & Petersen, 2012, lower than 0.35 mm) across the three fMRI acquisitions used in our study.

### MRI Data Acquisition

MRI data were acquired on a 3 Tesla Trio TIM whole-body scanner (Siemens, Erlangen, Germany), equipped with a 12-channel head coil. Functional BOLD images were acquired from a T2*-weighted gradient echo-planar imaging sequence (repetition time = 2,000 ms, echo time = 28 ms; field of view = 205 × 205 mm [matrix size = 64 × 64], slice thickness = 3 mm [no gap]; and flip angle = 90°). For anatomical coregistration of the fMRI images, a high-resolution T1-weighted image per subject was also acquired (MPRAGE, repetition time = 2,100 ms, echo time = 3.29 ms, inversion time = 1,100 ms, flip angle = 8°, field of view = 256 mm × 208 mm [matrix size: 256 × 208], slice thickness = 1 mm with no gap).

### Tasks

We used two tasks that involved processing conflicting information and response inhibition. Both tasks consisted of four blocks that defined a congruent information condition, interleaved with four blocks of trials where the participant received incongruent information. Both task conditions had a duration of 52–60 s and were preceded by a variable 10–17-s fixation block. In total, each task had a duration of 9 min and 20 s.

In the color-word Stroop task, participants had to select one of four identifier words using a response glove (e.g., thumb button 1 = identifier word on the far left, etc.), such that its name indicates the color of target words located in the center of a screen. During the congruent trials, the four identifier words were all in the same color as the target words. In incongruent trials, identifier words had all different colors, and the option to select was in a color incongruent with the target words. This kind of task usually evokes a brain response that activates regions in the anterior insula, parietal cortex, basal ganglia, thalamus, and cerebellum; while deactivating areas that belong to the so-called default-mode network (see Figures 6A, 6B, and 6C).

In the MSIT, which corresponded here to a modification from the original task version (Bush & Shin, 2006), participants had to select one of three numbers such that it differed from the other two by pressing buttons on the glove, where each button matched a number on the screen (thumb button 1 = number 1, etc.). During congruent trials, the targets’ position matched that on the glove, whereas during incongruent trials this position did not match the glove’s button location. This task elicits a brain pattern response that is largely similar to that in the Stroop task (see Figures 6D, 6E, and 6F and Sheu et al., 2012 for more details on the MSIT and the Stroop task).

In incongruent conditions of *both* tasks, accuracy was titrated to ∼60% by altering intertrial intervals; that is, consecutive accurate choices led to shortened intertrial intervals. To control for motor response differences between conditions in both tasks, the number of trials in the congruent condition was yoked to the number completed in the incongruent condition. Yoking was implemented by (1) administering an incongruent block first and (2) presenting congruent condition trials at the mean intertrial interval of the preceding incongruent block.

Finally, we also used a 5-min resting-state scan, during which the participants were told to keep their eyes open.

### Preprocessing

Data were preprocessed using fMRIprep (Esteban et al., 2019), a standard toolbox for fMRI data preprocessing that provides stability to variations in scan acquisition protocols, a minimal user manipulation, and easily interpretable, comprehensive output results reporting. First, anatomical data preprocessing was performed, including bias-field correction, skull-stripping, brain extraction and tissue segmentation, and surface reconstruction. It was then followed by functional data preprocessing, which included reference image estimation, head-motion parameter estimation, slice time correction, susceptibility distortion correction via a nonlinear registration (“Fieldmap-less” option of the toolbox), spatial normalization, and confounds estimation.

### Functional Correlation (Edge) Analysis

**Figure 7.**
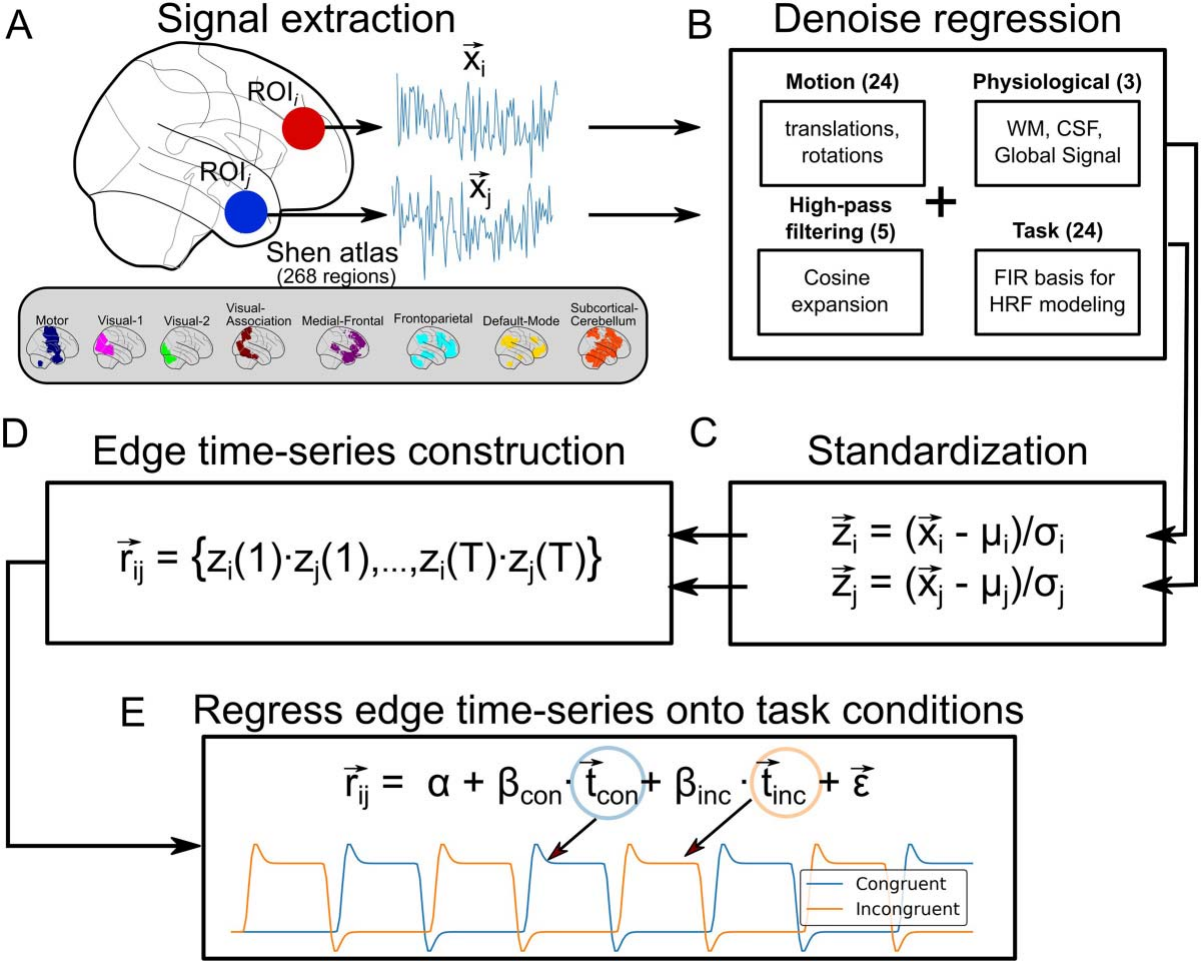
Estimation of intrinsic and task-related functional correlations. For a given pair of regions in the Shen atlas (consisting of 268 regions), the average signal within them was first computed (A). The time series were then denoised (B) and standardized (C). Subsequently, they were multiplied component-wise (D). Finally, the resulting temporal profile was regressed onto a design matrix to model intrinsic (intercept term) and task-related functional correlations (E).

We estimated task-based functional correlations using the edge, or cofluctuation, time series proposed in Faskowitz et al. (2020) and Zamani Esfahlani et al. (2020). One of the advantages of using this edge time series measure is that the procedure for estimating patterns of task-based functional correlation is by nature the same as that in GLM-based activation analyses but simply changing the outcome variable. A sketch of this full estimation procedure can be found in Figure 7. We first (Step A) reduced the spatial resolution of the preprocessed time series by computing the voxel-wise average signal within each region of interest (ROI) in a 268-parcel atlas (Shen, Tokoglu, Papademetris, & Constable, 2013). Following Finn et al. (2015), each of these regions was also identified to a specific intrinsic functional connectivity network: motor, visual-1, visual-2, visual-association, medial-frontal, frontoparietal, default mode, and subcortical-cerebellum. Then, let ${\vec{x}}_{i}$ ≡ {*x*_*i*_(1), …, *x*_*i*_(*T*)} be the time series of *T* scans (the full-scan sequence) for a given parcel *i* in such atlas. Each of these parcellated time series were subsequently (Step B) denoised by means of a linear regression model, in a single step that prevents artifacts from being reintroduced in the data (Lindquist, Geuter, Wager, & Caffo, 2019), in order to remove effects from motion (24 parameters that included 3 translations, 3 rotations, their derivatives, and the square of all these terms), the average white matter signal, the average CSF signal, the average brain signal, periodic oscillations greater than 187 s (5 cosine terms), and task activations (24 terms). This last set of regressors consisted of 12 finite impulse response (FIR) terms per task condition (congruent and incongruent) to flexibly model a hemodynamic response function (HRF) of about 24 s to external stimuli and that was included so as to avoid systematic inflation of functional correlations produced by task activations (Cole et al., 2019). The resulting denoised ROI time series were standardized (Step C), that is, ${\vec{z}}_{i}=\frac{{\vec{x}}_{i}-\mu }{\sigma }$, and then used to generate the edge time series ${\vec{r}}_{ij}$ (Step D) as the component-wise product between pairs of standardized time series, that is, ${\vec{r}}_{ij}$ = {*z*_*i*_(1) · *z*_*j*_(1), …, *z*_*i*_(*T*) · *z*_*j*_(*T*)}. At this point, if we summed these components and divided by *T* – 1, we would obtain the Pearson correlation coefficient that usually represents the static functional connectivity between BOLD time series—that is, each edge time series can be interpreted as a temporal decomposition of a functional connection (correlation) into its framewise contributions. Instead, we continued working on these edge time series as response variables in a general linear model (Step E) in order to estimate intrinsic and task-dependent functional correlation profiles. To this end, the input design matrix included an intercept term and a set of regressors for each task condition (congruent and incongruent), which comprised a boxcar function convolved with the usual double gamma hemodynamic response function and its temporal and dispersion derivatives. Although it is not clear that the usual hemodynamic response function also takes place in the edge time series, we decided to assume it for pipeline compatibility with the activation (node) analysis (see next subsection). Nevertheless, it is important to note that we repeated the same analytical pipeline considering just boxcar regressors (i.e., without including the hemodynamic response convolution), and found no substantial changes in the functional correlation profiles. Because of the considerable duration of each task condition, the effects triggered by the convolution with the hemodynamic response function, which mainly happen at the beginning and end of each task block, are dampened when averaging across all time points.

Prior to any statistical analysis, time series on both sides of the regression model were prewhitened through a first-order autoregressive model in order to account for the temporal autocorrelations. As mentioned earlier, we assumed this standard procedure for dealing with temporal autocorrelations in order to have the same statistical pipeline as that in the activation analysis (see next subsection). Future studies should investigate the most appropriate procedure for accounting for autocorrelations using edge time series. After this first-level estimation, task-based network changes were computed as contrasts of parameters and subsequently used to assess edge-wise group-level effects by means of a one-sample *t* test. Statistical inference at a usual 0.05 significance level was finally performed, after correcting the family-wise error (Holm-Bonferroni procedure) due to multiple testing. All of these statistical analyses of the edge time series were carried out using Nilearn 0.7 (Abraham et al., 2014).

### Activation (Node) Analysis

We also analyzed the preprocessed BOLD images at the node level, which involved estimating brain activation changes during the different task conditions. Such analyses are usually carried out at the voxel level. However, in order to keep the same resolution as that of the edge-level results, brain activations were estimated at the region level using the same parcellated BOLD time series.

For this analysis, we again employed a GLM with the parcellated BOLD time series as response variables and a design matrix that included the same set of task regressors used in the edge-wise analyses, as well as the same covariates that were regressed out prior to this, that is, the 24 motion parameters (K. J. Friston, Williams, Howard, Frackowiak, & Turner, 1996), the cosine terms to account for oscillation effects greater than 187 s, the average signal within white matter tissue, the average signal within CSF tissue, and the average signal within the whole brain. We considered this last regressor, not common in brain activation analyses, for consistency again with the edge-level analyses (see previous section). Group-level effects were similarly assessed using a one-sample *t* test.

### Generalized Psychophysiological Interaction

As a part of our sanity check pipeline, we compared the functional correlation analysis using the aforementioned edge time series approach with a model of generalized psychophysiological interactions (PPI), which is a standard approach for estimating task-dependent functional connectivity changes (McLaren, Ries, Xu, & Johnson, 2012) and is based on a general linear model of task-moderated temporal association between pairs of brain units. Specifically, for a given pair of BOLD time series ${\vec{x}}_{i}$ and ${\vec{x}}_{j}$, such a model includes one of them as the response variable and as inputs the other BOLD time series, the group of task regressors, the interaction terms between these task regressors and the input BOLD time series, and the possible confounders to consider in the model. In our case, the generalized PPI model can be written as follows:

$${\vec{x}}_{i}=\alpha +\beta_{ij}^{bck}\cdot {\vec{x}}_{j}+\beta^{\text{task}}\times 𝒯+\beta_{ij}^{ppi}\times {𝓘}_{i}+\beta^{\text{cov}}\times 𝒞+\vec{\epsilon },$$ (1)

where 𝒯 is a matrix whose columns are the HRF convolved boxcar congruent and incongruent time profiles and their derivatives and dispersion terms; 𝓘_*i*_ is the matrix with the PPI terms from each condition, that is, the interaction term between each task condition’s time profile and the input time series ${\vec{x}}_{i}$; and 𝒞 is a matrix with the different covariates to include in this model, which in our case comprised the 24 motion parameters, the average white matter signal, the average CSF signal, the average brain signal, and cosine expansion for a 187-s high-pass filtering. Once all the parameters in this model were estimated, task-based functional connectivity changes were evaluated by contrasting the incongruent and congruent PPI estimations, and their effect at the group level was assessed using a one-sample *t* test. In this way, a matrix of estimated task-based functional connectivity changes can be constructed. However, since a PPI model yields nonsymmetrical matrices, we symmetrized them by averaging their corresponding upper and lower triangular elements as done in Di, Reynolds, and Biswal (2017), which enabled direct comparison with the functional connectivity profiles obtained from the edge time series approach.

## SUPPORTING INFORMATION

Supporting information for this article is available at https://doi.org/10.1162/netn_a_00354.

## AUTHOR CONTRIBUTIONS

Javier Rasero: Conceptualization; Data curation; Formal analysis; Investigation; Methodology; Software; Supervision; Visualization; Writing – original draft; Writing – review & editing. Richard Betzel: Supervision; Writing – review & editing. Amy Isabella Sentis: Supervision; Writing – review & editing. Thomas E. Kraynak: Supervision; Writing – review & editing. Peter J. Gianaros: Funding acquisition; Resources; Supervision; Writing – review & editing. Timothy Verstynen: Conceptualization; Methodology; Project administration; Supervision; Writing – original draft; Writing – review & editing.

## FUNDING INFORMATION

Peter J. Gianaros, National Heart, Lung, and Blood Institute (https://dx.doi.org/10.13039/100000050), Award ID: P01 HL040962. Peter J. Gianaros, National Heart, Lung, and Blood Institute (https://dx.doi.org/10.13039/100000050), Award ID: R01 HL 1089850.

## CODE AND DATA AVAILABILITY

The code used to generate all the analyses and results can be found in https://github.com/CoAxLab/cofluctuating-task-connectivity (Rasero, 2023).

## Supplementary Material



## TECHNICAL TERMS

Functional MRI: MRI sequence that measures indirect brain activity as changes associated with blood flow.
BOLD: Blood oxygen level–dependent. Signal that reflects changes in deoxyhemoglobin triggered by localized changes in brain blood flow and blood oxygenation.
Edge time series: A time series that arises as the frame-to-frame multiplication of a given pair of brain regions’ standardized BOLD activity.
Functional connectivity: The network where each link or interaction quantifies the statistical dependency between pairs of brain regions’ activity over time.
Parcellation: Partition of the brain’s gray matter into nonoverlapping macroscopic ensembles, referred to as parcels or regions of interest, using a predetermined criterion.
Generalized psychophysiological interactions: A linear regression model that includes an interaction term between an external task stimuli and the BOLD activity of a given region or voxel.
Task activation: Average brain activity at the voxel or region level. It is usually calculated as the difference between the average brain activity during a challenging task condition and the average brain activity during an easy task condition.
Resting-state fMRI: A type of functional magnetic resonance imaging task where the subject is instructed to simply stay awake.

## Supporting Information

https://github.com/CoAxLab/cofluctuating-task-connectivity
