# Supplementary material for: Similarity in evoked responses does not imply similarity in macroscopic network states
Source: Netw Neurosci. 2024 Apr 1;8(1):335–54. doi: 10.1162/netn_a_00354 (PMC11073549; doi:10.1162/netn_a_00354)
Supplement: Supplementary file 1 [file netn-8-1-335-s001.pdf]

SUPPORTING INFORMATION

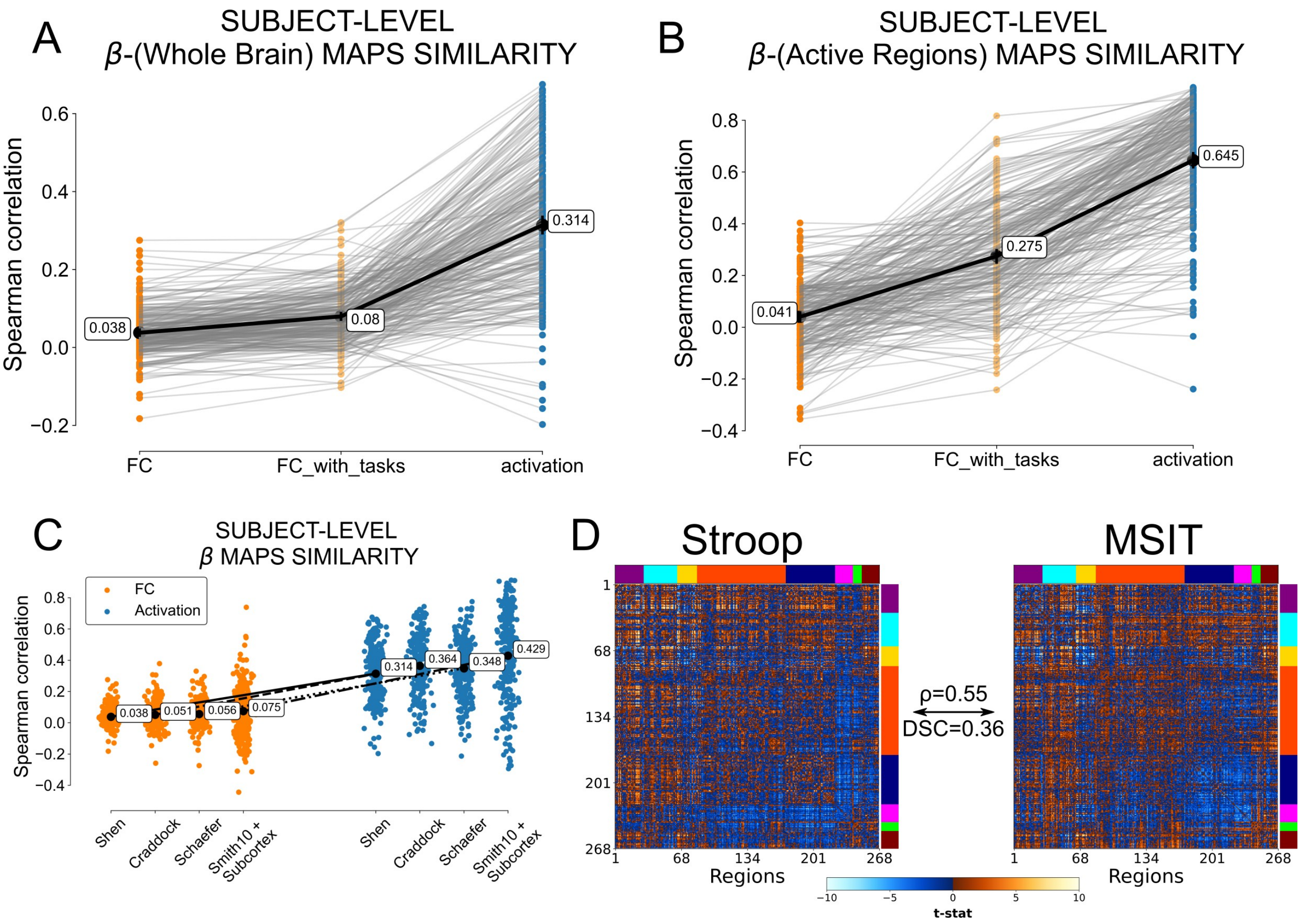

Supplementary Figure 1. **Replication analyses.** A) Same subject-level results as in Fig. 5F, but including the case where task stimuli were not regressed out prior the edge time series formation (”FC_with_tasks”). B) Same as A), but concentrating on the subset of regions with greatest task-evoked responses (group-level incongruent-vs-congruent absolute effect sizes, i.e. Cohen’s d, in activation larger than 0.8 in both tasks) C) At the subject level, between-task Spearman’s ρ correlation from brain activation (blue points) and task-based functional correlation (FC, orange points) β profiles for the Shen, Craddock, and Schaefer parcellations, as well as for a customized parcellation combining 10 ICA-based major areas and 7 subcortical regions from the Oxford-Harvard atlas. D) PPI-based incongruent-vs-congruent functional connectivity t-stat matrices for both Stroop and MSIT, and the similarity (Spearman’s ρ and DSC) between them.

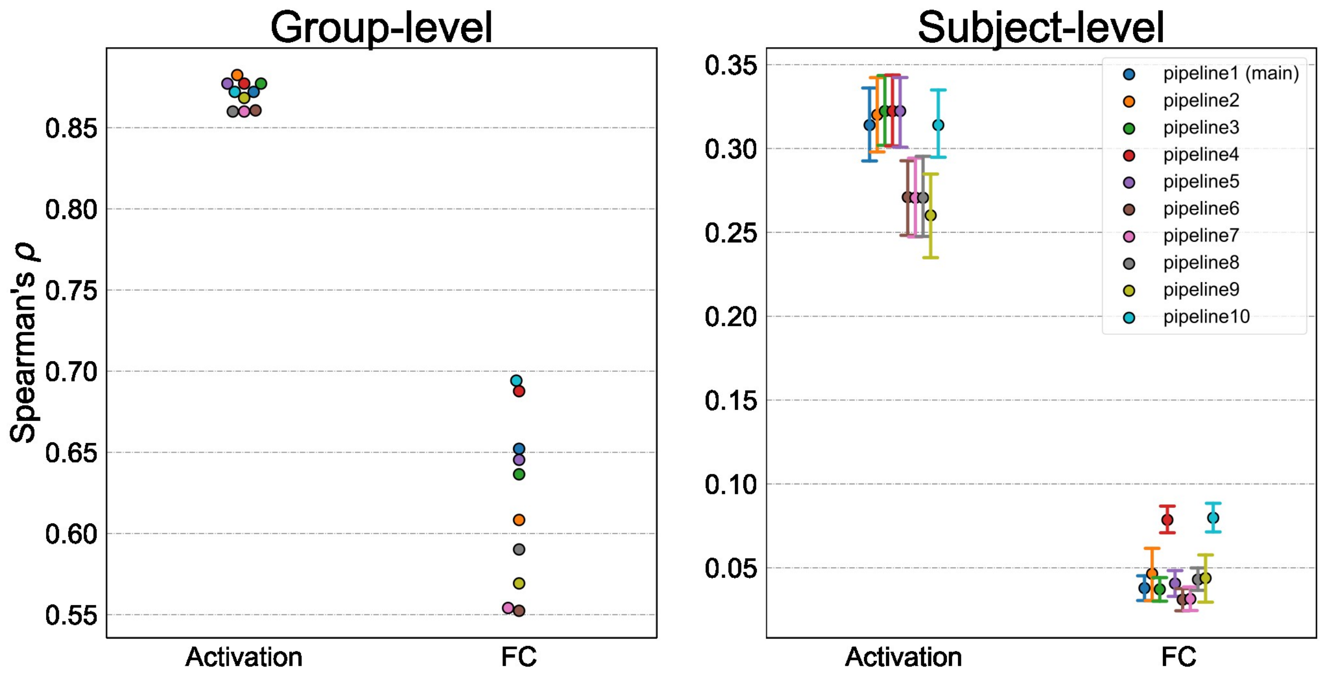

Pipeline \ Step	Task denoising	Global signal regression	HRF regressors	zscore time series	Prewhiten
1 (main)	FIR	✓	Canonical		✓
2	FIR		Canonical		✓
3	FIR	✓	Canonical	✓	✓
4	None	✓	Canonical	✓	✓
5	Canonical	✓	Canonical	✓	✓
6	Boxcar	✓	Boxcar	✓	✓
7	Boxcar	✓	Boxcar	✓	
8	None	✓	Boxcar	✓	
9	None		Boxcar	✓	
10	None	✓	Canonical		✓

Supplementary Figure 2. **Similarity results with different pipeline options.** Different pipelines were tested with five configurable steps. ”Task denoising” denotes the model used to remove task activity while building the edge time series (FIR, Canonical, or None if no task signal removal). ”Global Signal regression” indicates whether the global signal was removed. ”HRF regressors” indicates the task condition profiles for the first-level estimators. We also considered zscoring the outcome variables in the GLM model (”zscore time series” column), and prewhitening or not the residuals (”Prewhiten” column).

FIR≡ Finite Impulse Response basis, HRF≡ Hemodynamic Response Function. Canonical HRF includes the usual double gamma hemodynamic response function and its temporal and dispersion derivatives.
